# Comparison of Hemodynamic Effects of Dobutamine and Ephedrine Infusions in Isoflurane-Anesthetized Horses

**DOI:** 10.3390/vetsci10040278

**Published:** 2023-04-06

**Authors:** Sergio Grandisoli Garcia Filho, Felipe Silveira Rego Monteiro de Andrade, Rosana Souza Thurler dos Santos, Lucas Alaião Gonçalves, Marco Aurélio Amador Pereira, Anderson Fernando de Souza, Aline Magalhães Ambrósio, Denise Tabacchi Fantoni

**Affiliations:** Department of Surgery, School of Veterinary Medicine and Animal Science, University of São Paulo, 87 Professor Orlando Marques Paiva Ave., São Paulo 05508-270, SP, Brazil

**Keywords:** hypotension, sympathomimetics, cardiac output, vasoactive, inotropic

## Abstract

**Simple Summary:**

Transanesthetic hypotension is frequent in horses undergoing general inhalation anesthesia and can trigger severe postanesthetic complications. Consequently, the appropriate treatment must be instituted quickly; however, there are few studies in the species that help in the choice of the sympathomimetic drug when the inhaled agent used is isoflurane. The present study aimed to compare the cardiovascular effects of ephedrine and dobutamine infusions in healthy horses. Thirteen healthy horses were monitored with a catheter in the pulmonary artery, measuring serum lactate and troponin I, and parameters of ventilation and oxygenation. We identified that both drugs are effective in the treatment of hypotension in horses anesthetized with isoflurane.

**Abstract:**

The objective of this study was to compare the hemodynamic effects of dobutamine and ephedrine during the management of anesthesia-related hypotension in healthy horses. Thirteen horses underwent general anesthesia with isoflurane and were randomly divided into two different groups, one of which received a dobutamine constant rate infusion (CRI) (1 µg/kg bwt/min) and the other received an ephedrine CRI (20 µg/kg bwt/min) when hypotension (<60 mmHg) was identified, following up to 15 min after the blood pressure reached 70 mmHg. All horses were equipped with a pulmonary artery catheter and a peripheral artery catheter, and multiparameter monitoring commenced as soon as they were under mechanical ventilation. Hemodynamic parameters were recorded, while tissue perfusion markers (peripheral oxygen saturation, arterial oxygen partial pressure, arterial carbon dioxide partial pressure, arterial pH, arterial plasma bicarbonate concentration, arterial oxygen saturation, mixed venous oxygen saturation, mixed venous oxygen content, arterial oxygen content, arteriovenous oxygen difference, oxygen delivery index, oxygen consumption index, and oxygen extraction ratio), serum lactate concentration, and troponin I concentrations were analyzed before the start of infusions (T0), when the blood pressure reached 70 mmHg (T1), and 15 min after T1 (T2). The time to restore the arterial pressure was similar in both groups (*p* > 0.05); however, the heart rate was higher in the ephedrine group (*p* = 0.0098), and sinus bradyarrhythmia occurred in the dobutamine group. Furthermore, both experimental protocols increased cardiac output (*p* = 0.0012), cardiac index (*p* = 0.0013), systemic vascular resistance (*p* = 0.008), systemic vascular resistance index (*p* < 0.001), and ameliorated perfusion markers. In the dobutamine group, the pulmonary artery wedge pressure (*p* < 0.001) and systolic index (*p* = 0.003) were elevated, while the arteriovenous oxygen difference was reduced in the ephedrine group (*p* = 0.02). Troponin I was used as a myocardial injury indicator, and did not differ between moments or between groups (*p* > 0.05). We concluded that both drugs were effective and safe to treat anesthetic hypotension under the conditions of this study.

## 1. Introduction

Hypotension can be defined as low arterial blood pressure and is the most frequent perianesthetic complication observed after inhalation anesthesia in horses [1] and dobutamine is still the most often utilized drug in its management [2]. This agent is a positive inotrope that acts predominantly on β1 receptors [3], and its use in healthy horses has been shown to be more effective in increasing blood pressure and tissue perfusion than the use of vasopressors with α1 adrenergic properties, such as norepinephrine or phenylephrine [4].

However, considering that vasodilation is the main cause of hypotension during isoflurane anesthesia [5], it is suggested that the use of adrenergic agonists with α1 and β1 effects, such as ephedrine, would be an alternative to dobutamine. Indeed, in human beings, ephedrine is widely used to treat anesthetic hypotension and is associated with a few side effects such as tachycardia and arrhythmias [3,6]. In horses, the use of ephedrine in repeated and/or higher doses (0.2 mg/kg) generated an increase in heart rate and arrhythmia in which the long-term characteristic of the drug proved to be unfavorable, as the normal sinus rhythm was re-established only after 30 min, evidence of the importance of further studies, mainly to establish the recommended doses [7].

In view of this, the objective of this study was to compare the hemodynamic effects of dobutamine and ephedrine infusions in the management of hypotension in horses under general inhalational anesthesia with isoflurane. We hypothesize that there are no differences between the drugs in increasing blood pressure and improving tissue perfusion in healthy horses anesthetized with isoflurane.

## 2. Materials and Methods

### 2.1. Animals

The Ethics Committee on Animal Uses of the University of São Paulo approved this research, protocol number 6277290316. Thirteen Arabian gelding horses aged 5 years old and with body weights (bwt) from 280 to 360 kg were used in the study. The animals belonged to the institution. All horses were deemed healthy after cardiopulmonary auscultation, rectal temperature measurement, mucous membranes inspection, complete blood count, and serum biochemistry profiling.

### 2.2. Anesthesia

All animals underwent fasting for 8 h with free access to water. Prior to the anesthetic procedure, clipping and antiseptic preparation of the skin over both jugular veins was performed, a 14-gauge catheter was placed in the right jugular vein for the subsequent administration of drugs and fluids, and 7F catheter introducers were placed into the left jugular vein. The horses were premedicated with detomidine (Dormiun V, Agener União, São Paulo, Brazil) (10 µg/kg bwt) intravenously (IV); ten minutes later, general anesthesia was induced with ketamine (Dopalen, Vetbrands, Jacareí, Brazil) (2.2 mg/kg bwt IV), diazepam (Compaz, Cristália, São Paulo, Brazil) (0.05 mg/kg bwt IV), and 10% guaifenesin (Eter Gliceril Guaiacol, Powervet, São Paulo, Brazil) (50 mg/kg bwt IV), which was followed by tracheal intubation. The animals were positioned in dorsal recumbency, and anesthesia was maintained with isoflurane (Isoforine, Cristália, São Paulo, Brazil) (the end-tidal was maintained between 1–1.5%) diluted in oxygen (FiO_2_ = 70%) in a closed circuit to reach the third stage of anesthesia according to Guedel (the pupil in the ventromedial position and with medium size, palpebral reflex depressed, corneal reflex mildly depressed, respirations/heart rates normal or minimally depressed) [8]. A side stream, nondispersive, infrared gas analyzer (PoetIQ, Critical Care Systems Inc. Winsconsin, WI, USA) was used to monitor the inspired fraction of oxygen, carbon dioxide, and isoflurane, which was not altered throughout the experiment. Volume-controlled ventilation was instituted with an inspiratory:expiratory ratio of 1:2, a tidal volume of 14 mL/kg, and a respiratory rate of eight breaths/min (adjusted to maintain an end-tidal carbon dioxide of 35–45 mmHg). Lactated Ringer’s solution (Ringer lactato, JP Industria Farmacêutica S.A., Ribeirão Preto, Brazil) was administered at a rate of 10 mL/kg bwt/h during the anesthesia.

### 2.3. Instrumentation

Right after the animals were placed on mechanical ventilation, a 20-gauge catheter was placed in the transverse facial artery and was then coupled to the pressure transducer of the multiparametric monitor (DX 2020, Dixtal, Manaus, Brazil) and zeroed to the atmosphere at the height of the scapulohumeral joint to measure arterial pressure invasively. The same catheter was also used to collect arterial blood samples. A Swan–Ganz thermodilution catheter (Swan–Ganz; Edwards Lifesciences) was placed into the pulmonary artery and a second 6F balloon-tipped catheter was placed into the right atrium. Correct placement was confirmed by visual inspection of the pressure waveforms on the multiparameter monitor (DX 2020, Dixtal, Manaus, Brazil) and by observation of waveform flattening when the cuff was inflated with 1.5 mL of air. Heart rate (HR) and cardiac rhythm were also recorded through continuous electrocardiographic monitoring.

### 2.4. Study Design

The horses were randomized into two experimental groups that received different treatments when hypotension (MAP ≤ 60 mmHg) was detected, which was defined as the baseline moment (T0). Horses in the dobutamine group (n = 7) were treated with a dobutamine (Dobutatiston, Blau Farmacêutica, São Paulo, Brazil) (2.5 mg/mL solution) constant rate infusion (CRI) at an initial dose of 1 µg/kg bwt/min, which was increased by 1 µg/kg bwt/min every 5 min until a MAP of 70 mmHg was achieved or the maximum dose (5 µg/kg bwt/min) was reached, which was defined as the goal moment (T1). The infusion was maintained until the end of the evaluations (15 min after T1), which was defined as the after-moment (T2). Horses in the ephedrine group (n = 6) were treated with an ephedrine (Efedrin, Cristália, São Paulo, Brazil) CRI at an initial dose of 20 µg/kg bwt/min, which was maintained until the MAP increased to 60 mmHg; the dose was then decreased to 10 µg/kg bwt/min and maintained until a MAP of 70 mmHg was obtained (T1), which was followed by a second dose decrease to 5 µg/kg bwt/min that lasted until the end of the evaluations (T2).

### 2.5. Hemodynamic Parameters

With the multiparameter monitor (DX 2020, Dixtal, Manaus, Brazil), heart rate (HR), cardiac rhythm (DII lead electrocardiogram), systolic arterial pressure (SAP), mean arterial pressure (MAP), and diastolic arterial pressure (DAP) were monitored. Central venous pressure (CVP) and pulmonary arterial pressure (PAP) were recorded by connecting, respectively, the right atrium catheter and the pulmonary artery catheter to pressure transducers. The pulmonary artery wedge pressure (PAWP) was obtained by inflating the cuff of the pulmonary artery catheter with 1.5 mL of air. Finally, the cardiac output (CO) was measured (mean for three measures within 10% of variation) by the thermodilution method (infusion of glucose 5% (0.15 mL/kg at 0 °C) into the right atrium), and the cardiac index (CI) was calculated using a correction factor adequate for the species (k_bodyweight^2/3^, where k = 0.1) [9]. Based on these parameters, systolic index (SI), systemic vascular resistance (SVR), systemic vascular resistance index (SVRI), pulmonary vascular resistance (PVR), and pulmonary vascular resistance index (PVRI) were calculated with validated equations (Shoemaker, 2000):

*SI* = *CI* ÷ *HR*

*SVR* = (*MAP* − *CVP*) × 80 ÷ *CO*

*SVRI* = (*MAP* − *CVP*) × 80 ÷ *CI*

*PVR* = (*PAP* − *PAWP*) × 80 ÷ *CO*

*PVRI* = (*PAP* − *PAWP*) × 80 ÷ *CI*

### 2.6. Perfusion Markers

Serum lactate concentration was measured using a portable lactometer (Accutrend Plus, Roche, São Paulo, Brazil), using venous blood, and before initiating the dobutamine or ephedrine CRI at T0 and T2. Furthermore, the peripheral oxygen saturation (SpO_2_) was recorded by the multiparameter monitor’s pulse oximeter, which was placed on the tongue.

At T0 and T2, arterial blood samples were collected from the transverse facial artery catheter, and mixed venous blood samples were collected from the pulmonary artery catheter to measure the arterial oxygen partial pressure (PaO_2_), arterial carbon dioxide partial pressure (PaCO_2_), arterial pH (pHa), arterial plasma bicarbonate concentration (HCO_3_-), arterial oxygen saturation (SaO_2_), and mixed venous oxygen saturation (SvO_2_). Subsequently, the mixed venous oxygen content (CvO_2_), arterial oxygen content (CaO_2_), arteriovenous oxygen difference (C(a-v)O_2_), oxygen delivery index (DO_2_I), oxygen consumption index (VO_2_I), and oxygen extraction ratio (OER) were calculated.

### 2.7. Myocardial Injury Indicators

Venous blood samples were collected immediately before T0 and 4 h after T2 by jugular catheter. The samples were centrifuged at 3000 rpm for 5 min, and the serum was stored under −80 °C for posterior measurement of serum concentration of troponin I using an ELISA method (i-STAT cartridge Troponin (cTnI), Abbott Point of Care Inc. Haia, Holanda) [10,11].

### 2.8. Recovery of Horses from Anesthesia

After the procedure, the horses were taken to an appropriate anesthesia recovery stall and were immediately given xylazine (Equisedan, J.A. Saúde Animal, Patricínio Paulista, Brazil) (0.3 mg/kg) IV. Extubation was performed when the swallowing reflex was observed, and recovery was assisted with support ropes attached to the tail and to the halter.

### 2.9. Data Analysis

The normal distribution of data was verified using Shapiro–Wilk and Kolmogorov–Smirnov tests. To compare means between the moments within each group, one-way repeated measures analysis of variance (ANOVA) and Bonferroni post hoc tests were used. The Student’s *t*-test was used to compare groups regarding the time taken to reach the MAP goal. Two-sided comparisons were made. The results were considered significant if *p* < 0.05. All statistical analyses were performed using GraphPad Prism 7.02 software.

The power analysis carried out a posteriori was 92%, indicating that the sample size was adequate. For this, we used the *t*-tests—Means: the difference between two independent means (two groups) from GPower software (version 3.1.9.7) [12], considering the effect size of 1.84, which was estimated from the heart rate data at T1 and a probability of alpha error of 0.05.

## 3. Results

Hypotension was detected in all animals at the beginning of inhalational anesthesia as soon as invasive pressure measurements were implemented, which took around 10 min to complete. The mean time to reach the target MAP (70 mmHg) was 8.7 ± 2.3 min in the dobutamine group and 6.7 ± 1.2 min in the ephedrine group, which did not differ significantly (*p* = 0.47). All postanesthetic recoveries were without complications.

### 3.1. Hemodynamic Parameters

**Figure 1:**
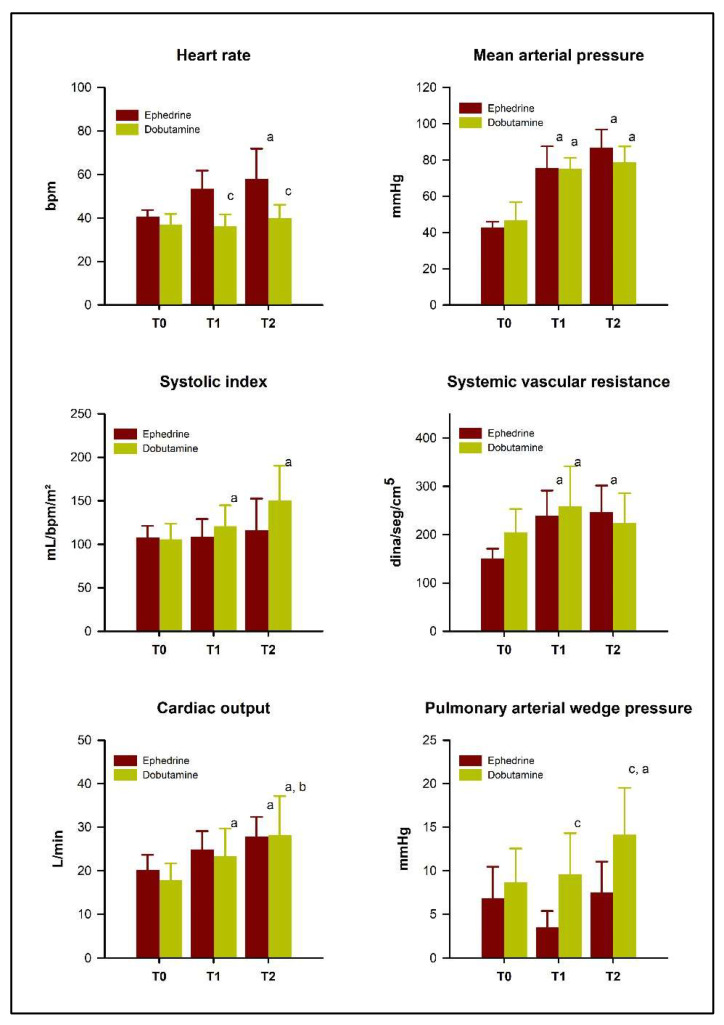
Changes in heart rate, mean arterial pressure, systolic index, systemic vascular resistance, cardiac output, and pulmonary arterial wedge pressure in 13 isoflurane-anesthetized horses treated with dobutamine or ephedrine, measured at the time of the detection of hypotension (T0), upon reaching a mean arterial pressure of 70 mmHg (T1), and 15 min after T1 (T2). Superscript letters indicate a significant difference (*p* < 0.05) from the basal moment (a), from goal moment (b), or from the other group in the same moment (c).

In the ephedrine group, HR (Figure 1) was higher at T1 (*p* = 0.003) and T2 (*p* = 0.02) than at T0, and the mean HR was higher in the ephedrine group than in the dobutamine group at T2 (*p* = 0.0098). However, 28.6% (2/7) of horses in the dobutamine group had sinus bradyarrhythmia during the infusion of the drug. Half of the horses treated with ephedrine (3/6) developed tachycardia (>40 bpm), while bradycardia (<28 bpm) was noted in 28.6% (2/7) of horses receiving dobutamine.

Both experimental protocols increased SAP, MAP (Figure 1), and DAP significantly (*p* < 0.05), as well as CO, CI, SVR, and SVRI (*p* < 0.05), without significant differences between groups (*p* > 0.05). The systolic index (SI) increased in the dobutamine group only (*p* = 0.003) and was higher at T1 and T2 than at T0 in that group; however, it did not differ significantly between groups at any moment (*p* > 0.05). CVP, PVR, PVRI, and PAP did not change with the use of either drug (*p* > 0.05). Nonetheless, the dobutamine group showed a PAWP increase (Figure 1), which was greater at T2 than at T0 and T1, and the PAWP values at T1 and T2 were also significantly higher in the dobutamine group compared with the ephedrine group (*p* < 0.001). The mean values of all hemodynamic parameters are listed in Table 1.

### 3.2. Perfusion Markers

**Figure 2:**
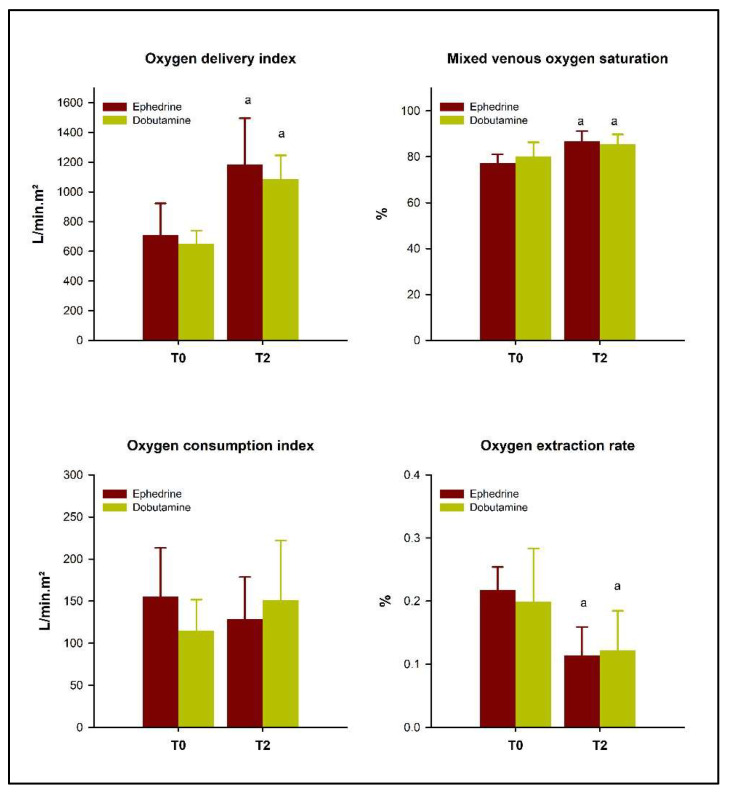
Changes in oxygen delivery index, mixed venous oxygen saturation, oxygen consumption index, and oxygen extraction rate in 12 isoflurane-anesthetized horses treated with dobutamine or ephedrine, at the time of the detection of hypotension (T0), and 15 min after reaching a mean arterial pressure of 70 mmHg (T2). Superscript letters indicate a significant difference (*p* < 0.05) from the basal moment (a).

Serum lactate was not different between groups or between moments (*p* > 0.05). The same was noted for SpO_2_, pH, HCO_3_-, PaO_2_, SaO_2_, CaO_2,_ and VO_2_I, none of which were altered by either drug. On the other hand, PaCO_2_ (Table 2), CvO_2_, SvO_2_, DO_2_I, and OER (Figure 2) changed in both groups; however, there was no significant difference between the treatments (*p* > 0.05). C(a-v)O_2_ increased in the ephedrine group only (*p* = 0.02), but there was no significant difference between groups. The mean values of all perfusion markers are listed in Table 2.

### 3.3. Myocardial Injury Indicators

The serum concentration of troponin I was 0 ng/mL in all animals before initiating the drug infusions. After anesthesia, the mean value was 0.026 ± 0.04 ng/dL for the ephedrine group and 0.038 ± 0.05 ng/mL for the dobutamine group, which was not significant and did not differ between groups or between moments (*p* > 0.05).

## 4. Discussion

MAP was restored to values greater than or equal to 70 mmHg within the same time period with the use of both dobutamine and ephedrine; however, side effects such as tachycardia and arrhythmias were observed. Moreover, the elevation of serum troponin I levels, and ST segment deviations were not observed in the study, which suggests that the administration of these drugs in the present scenario is probably not associated with cardiac ischemia [13].

Ephedrine is usually administered as a bolus as it is longer acting when compared to other sympathomimetic drugs [3] and, according to a study in dogs, tachyphylaxis after prolonged use can occur [14]. Nevertheless, a study in horses found that when the ephedrine infusion dose is kept constant, high values of blood pressure are observed [15]. Ephedrine acts directly as an agonist of α1, α2, β1, and β2 receptors, and indirectly facilitates the release of noradrenaline and inhibits the action of monoamine oxidase. This effect can be interrupted by the depletion of norepinephrine stores, leading to tachyphylaxis with its recurrent use [3]. In horses, as well as in dogs and guinea pigs, ephedrine biotransformation occurs mainly by demethylation into norepinephrine, which undergoes renal excretion in an unchanged form. This process occurs rapidly, suggesting that most of its effect is triggered by norepinephrine [16,17].

In the present study, tachyphylaxis was not observed since blood pressure was normalized and maintained at adequate levels throughout the evaluation period. The gradual decrease in the infusion rate allowed blood pressure to be restored without triggering episodes of hypertension.

Vasopressors can increase blood pressure without necessarily increasing cardiac output. Blood pressure is the product of cardiac output and SVR; therefore, tissue perfusion may not always be re-established by vasoconstriction [4,18]. Both agents increased cardiac output and SVR. Ephedrine is known to have both α and β-adrenergic activities, which corroborates our results and others in the literature [15,19]. However, dobutamine is a predominantly inotropic agent with some β2 effects that are responsible for a decrease in SVR [4,20]. The clinically utilized racemic mixture contains the (−) enantiomer, which has an important α1 action, and the (+) enantiomer, which acts predominantly on β1 and β2 receptors. However, the SVR is usually maintained because β2 and α1 effects are equivalent [21,22]. Even though it was transitory, the horses that received dobutamine showed an increase in SVR, suggesting that its effects on α1 receptors are not negligible, as discussed by Vries et al. [23] when observing an increase in blood pressure without elevation of cardiac index in horses treated with dobutamine. Although their mechanisms of action are different, both agents produced similar clinical effects regarding cardiovascular function.

The increase in SvO_2_ and DO_2_I as well as the stability of serum lactate levels observed in the study are probably due to the maintenance of adequate tissue perfusion. Even though these parameters do not reflect tissue perfusion directly, previous studies have shown that they are good indicators [23,24,25,26].

Both agents produced an improvement in hemodynamic parameters and tissue perfusion markers with a low rate of complications. Although it has been described that dobutamine can increase pulmonary artery pressure and pulmonary vascular resistance [5,27], these parameters did not elevate in the present study. However, there was a significant increase in PAWP only in horses treated with dobutamine, showing values higher than the reference range for the species [28]. This parameter is very important in the diagnosis of pulmonary hypertension in humans [29,30]. Therefore, the possibility of a PAWP increase during dobutamine administration should be considered in conditions associated with pulmonary hypertension, such as asthma [31].

Ephedrine administration led to tachycardia in half of the horses, reaching heart rate values higher than the physiological range for the species, which has been reported in the literature [7,20]. However, this effect is not consistent, as other studies have not observed tachycardia, even with ephedrine constant rate infusion [13,15,32]. On the other hand, two horses in the dobutamine group that needed a dose increase to 4 µg/kg bwt/min developed bradycardia after the blood pressure was restored. This effect is not uncommon; however, increments in the dobutamine dose are more frequently accompanied by heart rate increases and arrhythmias. It has been reported that high doses of dobutamine (4–10 µg/kg/min) have caused tachycardia [5,33] and tachycardia with atrioventricular conduction block [34].

Despite these alterations in heart rate, the administration of both dobutamine and ephedrine was not associated with cardiac ischemia since serum troponin I levels remained within the normal range [10,35]. Troponin I has been used as a marker of cardiac injury in horses, especially in racehorses, in which it peaks 4 to 6 h after myocardial injury [10]. In this study, samples were collected 4 h after the end of infusions and did not differ from the baseline moment, demonstrating that neither protocol used promoted injury to the myocardium. Slack et al. [35] and Rossi et al. [10] report the lack of standardization between the analysis methods and, consequently, the large range of normality reported for this species in the literature. The I-STAT assay has been shown to be efficient to measure troponin I in horses of different categories [36,37].

As limitations, we can highlight that a longer evaluation time than that carried out in this study could show the hemodynamic repercussions of greater exposure to drugs. The use of direct assessments of tissue perfusion, such as spectroscopy or spectral imaging by orthogonal polarization, would provide a qualitative and quantitative analysis of the microcirculation. Furthermore, the results of this study reflect the physiological response of small (260–380 kg bwt) and healthy horses, which can be limiting when extrapolating to larger animals (above 600 kg bwt) and/or with conditions that affect tissue perfusion.

## 5. Conclusions

This study found no difference between the two drugs. Our clinical observations suggest that they are both effective in treating hypotension in horses anesthetized with isoflurane and that both dobutamine and ephedrine increased the cardiac index and the peripheral tissue perfusion markers.

## Figures and Tables

**Table 1:** Mean changes (± standard deviation) of hemodynamic parameters under the influence of ephedrine or dobutamine infusion in 13 isoflurane-anesthetized horses at the time of the detection of hypotension (T0), upon reaching a mean arterial pressure of 70 mmHg (T1), and 15 min after T1 (T2).

Variables	Ephedrine			Dobutamine		
	T0	T1	T2	T0	T1	T2
SAP (mmHg)	68 ± 8	107 ± 11 ^a^	120 ± 9 ^a^	68 ± 12	114 ± 10 ^a^	117 ± 17 ^a^
DAP (mmHg)	31 ± 1	61 ± 12 ^b^	70 ± 14 ^b^	35 ± 9	59 ± 9 ^b^	60 ± 8 ^b^
CVP (mmHg)	2 ± 2	2 ± 4	2 ± 4	3 ± 1	5 ± 3	5 ± 4
PAP (mmHg)	9 ± 4	9 ± 4	12 ± 5	11 ± 6	14 ± 3	15 ± 2
CI (L/min/m^2^)	4.5 ± 0.7	5.5 ± 1	6 ± 1.1 ^a^	3.8 ± 0.9	5 ± 1.4 ^a^	6 ± 1.9 ^ab^
SVRI (dina.seg/cm^5^.m^2^)	682 ± 84	1085 ± 250 ^a^	1122 ± 268 ^a^	951 ± 241	1205 ± 433 ^a^	1041 ± 300
PVR (dina.seg/cm^5^)	19 ± 13	25 ± 10	31 ± 15	31 ± 17	34 ± 15	41 ± 15
PVRI (dina.seg/cm^5^/m^2^)	62 ± 30	105 ± 35	119 ± 78	36 ± 11	98 ± 59	72 ± 21

SAP: systolic arterial pressure, DAP: diastolic arterial pressure, CVP: central venous pressure, PAP: pulmonary artery pressure, CI: cardiac index, SVRI: systemic vascular resistance index, PVR: pulmonary vascular resistance, and PVRI: pulmonary vascular resistance index. Superscript letters indicate a significant difference (*p* < 0.05) from the basal moment (^a^), from goal moment (^b^).

**Table 2:** Mean changes (± standard deviation) of perfusion markers under the influence of ephedrine or dobutamine infusion in 13 isoflurane-anesthetized horses at the time of the detection of hypotension (T0), and 15 min after reaching a mean arterial pressure of 70 mmHg (T2).

Variables	Ephedrine		Dobutamine	
	T0	T2	T0	T2
Lactate (mmol/L)	2.6 ± 1.2	2.4 ± 1	2.7 ± 0.6	2.9 ± 0.6
pH	7.37 ± 0.04	7.38 ± 0.08	7.38 ± 0.04	7.34 ± 0.03
HCO_3_- (mmol/L)	26 ± 3	27 ± 4	28 ± 4	29 ± 2
SpO_2_ (%)	98 ± 1	97 ± 2	98 ± 1	97 ± 1
PaO_2_ (mmHg)	186 ± 30	194 ± 80	224 ± 43	226 ± 60
PaCO_2_ (mmHg)	46 ± 2.1	49 ± 2.8 ^a^	48 ± 2.8	43 ± 1.1 ^a^
SaO_2_ (%)	99 ± 0.7	99 ± 0.5	99 ± 0.2	99 ± 0.4
CaO_2_ (L/dL)	17.2 ± 3.6	20.7 ± 4	21 ± 4.9	21.9 ± 4.7
Ca-vO_2_ (mL/dL)	343 ± 93	209 ± 69 ^a^	267 ± 30	271 ± 142

HCO_3_-: bicarbonate ion, SpO_2_: peripheral oxygen saturation, PaO_2_: arterial oxygen partial pressure, PaCO_2_: arterial carbon dioxide partial pressure, SaO_2_: arterial oxygen saturation, CaO_2_: arterial oxygen content, Ca-vO_2_: arteriovenous oxygen difference. Superscript letters indicate a significant difference (*p* < 0.05) from the basal moment (^a^).

## Data Availability

The data presented in this study are available on request from the corresponding author.
